# Mortality rates in adults with severe congenital heart disease: insights from the National Congenital Heart Disease Registry of Australia

**DOI:** 10.1016/j.ijcchd.2025.100629

**Published:** 2025-10-17

**Authors:** Jason Chami, Calum Nicholson, David Baker, Rachael Cordina, Geoff Strange, David S. Celermajer

**Affiliations:** a Sydney Medical School, University of Sydney, Camperdown, NSW, 2006, Australia; b Heart Research Institute, 7 Eliza St, Newtown, NSW, 2042, Australia; c Royal Prince Alfred Hospital, Missenden Rd, Camperdown, NSW, 2050, Australia; d School of Medicine, University of Notre Dame Australia, 21 Henry St, Fremantle, WA 6160, Australia

**Keywords:** Adult congenital heart disease, Severe congenital heart disease, Single-ventricle physiology, Geographic disparities

## Abstract

**Background:**

Survival into adulthood with congenital heart disease (CHD) has improved, but premature mortality remains common. Long-term, multi-centre data are scarce. We used a validated complexity algorithm to select “severe CHD” patients from the National CHD Registry of Australia, quantifying mortality by ventricular physiology, residential remoteness and treating-centre location.

**Methods:**

From 70185 CHD patient records, we identified 6557 patients classified as “severe” by 2020 European Society of Cardiology guidelines, born from 1980 onwards and alive at 16 years old. We found 2118 single-ventricle patients, 3674 two-ventricle and 765 with insufficient information for classification. Residence was grouped into Major Cities, Inner Regional, and Outer Regional/Remote. Vital status follow-up was to December 31, 2023. Kaplan–Meier curves were used to compare survival.

**Results:**

Overall, 25-year survival was 96.9 % and 40-year survival was 89.0 %. Survival differed by physiology: single-ventricle survival was 96.4 % at 25 years and 88.2 % at 40 years, while two-ventricle survival was better, at 97.6 % at 25 years and 90.8 % at 40 years (p = 0.0015). Patients managed in Sydney/Melbourne fared better than those treated elsewhere, particularly after age 35 (25-year survival ∼97 % for both; 40-year survival 89.9 % vs 81.9 %; p = 0.027). Survival did not, however, vary with residential remoteness.

**Conclusion:**

Adults with severe lesions, particularly single-ventricle physiology, remain at high risk of premature mortality. Patients managed in Sydney/Melbourne had better survival, possibly reflecting the influence of long-established specialist ACHD programs, although residual confounding cannot be excluded. Urban-rural outcome parity suggests that Australia's centralised care model mitigates geographical barriers.

## Highlights

• We analysed 6557 adults with severe CHD using a national Australian registry.
• Conditional 40-year survival from age 16 was 89.0 % vs 98.7 % in the general population.
• Single-ventricle physiology carried a higher mortality risk than two-ventricle.
• Sydney/Melbourne centres excelled, though rural residence did not impair survival.

## 1 Introduction

Congenital heart disease (CHD) used to carry a very guarded prognosis, but improvements in paediatric cardiology and cardiac surgery have dramatically improved survival [1]. In recent decades, in fact, over 90 % of infants born with CHD have reached adulthood [2]. As a result, adults with CHD (ACHD) now outnumber children with CHD in many developed countries [3,4]. Despite this success, CHD outcomes continue to vary widely depending on the anatomical and physiological complexity of the underlying cardiac lesion. Severe (or “complex”) lesions (e.g. single-ventricle hearts or Eisenmenger syndrome) carry a significantly higher risk of early mortality compared to simpler defects [5,6]. There is therefore a pressing need to better understand and track outcomes in high-risk CHD patients as they age.

A major challenge in this endeavour is the wide variety of CHD lesions managed in specialist centres. Though CHDs are the most common birth defect as a group, this group consists of hundreds of rare anomalies with varied comorbidities and surgical approaches. Therefore, to enable meaningful research on such a disparate cohort, a) the data from multiple specialist centres must be combined to increase the sample size, and b) severity/complexity stratification systems are required that stratify lesions into mild, moderate and severe disease. Multiple severity classifications have been designed to correspond with prognosis and expected healthcare utilization, but as management and prognosis has changed, the rules for grouping lesions have had to be regularly updated, with the current European Society of Cardiology (ESC) guidelines just the latest in a long list [7,8]. Though these guidelines have transformed CHD research, they have largely required manual severity coding by expert clinicians, which is labour-intensive and prone to inconsistency [9]. As a result, many large CHD databases rely on simpler, outdated severity scores, or lack severity scoring at all [10].

The National CHD Registry of Australia was established to address these gaps by bringing together data from all CHD centres in Australia [11], tracking mortality via linkage with the Australian National Death Index and automatically classifying patients up-to-date severity scores using a novel algorithm. This algorithmic tool, validated against expert review, improves classification over diagnosis-only methods by incorporating procedural history, for example by recognizing that a patient who has had a Fontan procedure must have a functionally univentricular heart even if they are missing other diagnostic data [9,12]. Using this tool, we can reliably identify adults with “severe” CHD, enabling focused analysis of this growing subgroup.

Within complex CHD, single ventricle patients represent a well-recognised high-risk group, but to our knowledge no prior study has directly compared their adult survival outcomes to those of other complex CHD patients. Additionally, while geographic disparities in general cardiovascular health are well-known, with rural communities often facing worse outcomes, it remains unclear whether CHD outcomes differ by rurality, given the highly specialised and centralised care rural patients usually travel to receive in the capital cities of Australia and New Zealand. Finally, we hypothesised that patients treated in Sydney or Melbourne, which have the largest, oldest specialised ACHD services, and long-standing access to advanced cardiothoracic surgery, may have better outcomes than those patients seen in other cities. In this study, we report mortality rates from our registry, examining differences in survival in patients with single-versus two-ventricle anatomy, between patients being treated in Sydney or Melbourne versus other cities, and between those living in major cities versus regional areas.

## 2 Methods

### 2.1 Ethics and consent

Ethics approval for the National CHD Registry of Australia has been granted by the Human Research Ethics Committee at Royal Prince Alfred Hospital (2019/ETH07472). A waiver of consent is in place for the analysis of retrospective data.

### 2.2 Data collection

#### 2.2.1 Data source

This study utilised the National Congenital Heart Disease Registry of Australia, a multi-institutional registry that collates clinical data from individuals with CHD across all major congenital cardiac centres in Australia. The registry captures demographics, detailed diagnosis codes using the European Paediatric Cardiac Code [13], and surgical and catheter intervention history, with linkage to national death registries and various healthcare utilization registries ensuring comprehensive follow-up [10].

#### 2.2.2 Severity and physiology classification

We used a novel, validated algorithm to classify patients into mild, moderate and severe disease according to 2020 ESC guidelines [7]. This algorithm has been previously described [9,12]; in summary, it combines a list of diagnoses with associated procedures and compares the combined list to a large lookup table developed to match ESC guidelines. For example, a patient with a diagnosis code for transposition of the great arteries would be classified as “moderate” CHD if they had an arterial switch procedure, “severe” if they had an atrial switch procedure, and “unknown” if there was no procedural information available. The algorithm can sometimes also use procedural information without corresponding diagnostic data. For example, a patient with a recorded Fontan operation can be classified as “severe” CHD, even if a specific single-ventricle diagnosis code was not present, because the Fontan procedure itself implies the existence of complex univentricular CHD. This algorithm demonstrates accuracy above 99 % when compared to CHD specialists presented with the same diagnostic and procedural data—for this study we considered the classification as definitive, and only included patients categorised as “severe” by our algorithm (n = 6557). Because of the complex interplay of diagnostic and procedural codes within the algorithm, it is impossible to say whether a particular patient was classified by diagnosis or by procedure, as all patients with both sets of data are classified by both in tandem.

For this study, we augmented the algorithm with a lookup table that used the list of diagnoses and procedures to classify the cohort of complex adult patients as single-ventricle (e.g. hypoplastic left heart syndrome, tricuspid atresia, etc.; n = 2118), two-ventricle (e.g. transposition of the great arteries with atrial switch, pulmonary atresia with VSD, etc.; n = 3674), or unknown (n = 765).

#### 2.2.3 Variables of interest

Along with diagnostic and procedural data, we extracted age, sex, clinic location and residence location. Residence location was categorised according to the Australian Statistical Geography Standard Remoteness Areas: Major Cities, Inner Regional, Outer Regional, Remote and Very Remote, with the latter three categories grouped together for purposes of statistical analysis. As adult hospital care in Australia begins at age 16, we extracted only patients who reached 16 years of age. As CHD surgery was not routinely offered to children with complex CHD in Australia before 1980, we chose to exclude patients born before January 1, 1980, to avoid including outcomes from an era of CHD management that is no longer representative of the standard of care.

### 2.3 Data analysis and statistical methods

The primary outcome was all-cause mortality. Survival was modelled on attained age, with delayed entry at age 16 to reflect the transition from paediatric to adult follow-up. Thus, a survival estimate at “25 years” corresponds to survival from age 16 to 25, and a survival estimate at “40 years” corresponds to survival from age 16 to age 40. Follow-up time was calculated until death or December 31, 2023, the most recent time that the Australian National Death Index was queried. We constructed Kaplan–Meier curves for the overall severe CHD cohort, and for three key covariates of interest: single-versus two-ventricle physiology, treatment in Sydney/Melbourne versus other cities, and geographic remoteness. For single-versus two-ventricle comparisons, patients that could not be confidently classified into either category were excluded. For geographical comparisons, patients who resided in areas with multiple possible remoteness classifications were excluded. All survival curves were compared using log-rank tests. A multivariate Cox proportional hazards model was generated with the following covariates: physiology (single-versus two-ventricle), treatment location (Sydney/Melbourne versus other city), residential remoteness (major city versus inner regional versus outer regional/remote), sex, presence of genetic comorbidities, and birth decade. Proportional hazards assumption testing for each variable was conducted using Schoenfeld residuals tests. Data management and analysis followed the Strengthening the Reporting of Observational Studies in Epidemiology (STROBE) guidelines for cohort studies.

## 3 Results

### 3.1 Overall cohort characteristics

**Fig. 1:**
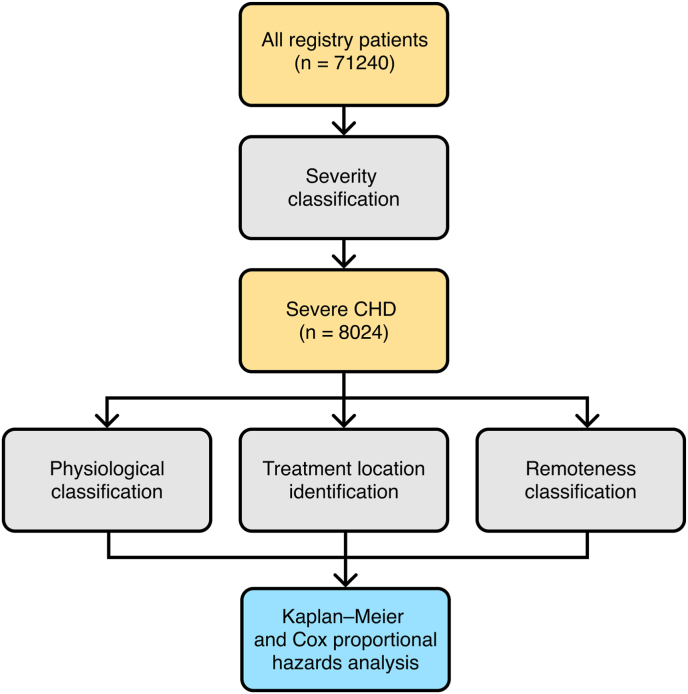
Flowchart of patient selection and analysis.

Patient selection is summarised in Fig. 1 and baseline characteristics are summarised in Table 1. The prevalences of a selection of common severe CHD diagnoses are presented in Table 2. Common diagnoses included transposition of the great arteries (n = 1955), pulmonary atresia (n = 1200), and double-outlet right ventricle (n = 987). By design, all patients were classified as “severe” according to 2020 ESC guidelines; a minority (n = 603; 9.2 %) had coexisting genetic syndromes (e.g. Down or DiGeorge syndromes). The most commonly-performed procedures in this cohort are presented in Table 3. Common procedures included systemic-to-pulmonary arterial shunts (n = 1795), Fontan-type procedures (n = 1096), bidirectional superior cavopulmonary (Glenn) anastomoses (n = 848), and ventricular septal defect closures (n = 846).

**Table 1:** Cohort characteristics.

Characteristic	n (%)
**Total**	6557 (100 %)
**Sex**	
Male	3651 (56 %)
Female	2893 (44 %)
Not Stated	13 (0.2 %)
**Physiology**	
Single-Ventricle	2118 (32 %)
Two-Ventricle	3674 (56 %)
Unknown	765 (12 %)
**Clinic Location**	
Sydney/Melbourne	5923 (90 %)
Other	634 (10 %)
**Remoteness**	
Major Cities	3590 (55 %)
Inner Regional	844 (13 %)
Outer Regional/Remote	310 (5 %)
Multiple	511 (8 %)
Unknown	1302 (20 %)
**Genetic Syndromes**	
Down Syndrome	428 (6.5 %)
DiGeorge Syndrome	133 (2.0 %)
Edwards Syndrome	12 (0.2 %)
Turner Syndrome	8 (0.1 %)
Patau Syndrome	2 (0.03 %)
Unspecified Chromosomal Abnormality	20 (0.3 %)

**Table 2:** A selection of severe congenital heart disease diagnoses and their prevalence in our cohort. Note that patients may have multiple diagnoses, for example “functionally univentricular heart (unspecified)” as a result of a procedure, as well as “hypoplastic left heart syndrome”.

Diagnosis	N
Transposition of the great arteries	1955
Pulmonary atresia	1200
Double-outlet right ventricle	987
Functionally univentricular heart (unspecified)	833
Hypoplastic left heart syndrome	438
Congenitally-corrected transposition	326
Tricuspid atresia	294
Interrupted aortic arch	263
Truncus arteriosus	245
Eisenmenger syndrome	94

**Table 3:** The most common procedure performed on our cohort of 6557 patients with severe congenital heart disease, summarised by broad EPCC category. For example, the Modified Blalock interposition shunt and the central systemic-to-pulmonary arterial interposition shunt have been grouped together under the category of “systemic-to-pulmonary arterial shunt procedures.”

Procedures	N
Systemic-to-pulmonary arterial shunt procedure	1795
Fontan type procedure	1096
Bidirectional superior cavopulmonary (Glenn) anastomosis	848
Ventricular septal defect closure	846
Aortic arch repair	574
Conduit construction procedure	506
Patent ductus arteriosus closure	503
Norwood type procedure	442
Pulmonary arterioplasty/reconstruction	401
Balloon atrial septostomy by pull back (Rashkind)	386
Interatrial communication creation/enlargement	302
Pulmonary artery band	292
Tetralogy of Fallot repair	284
Atrial septal defect secundum closure	270
Right ventricular outflow tract procedure	254

In total, around 185,000 patient-years of follow-up were under analysis. During this period, there were 313 deaths in the cohort. As survival was modelled on attained age with delayed entry at age 16, our “25 year” estimates reflect survival from age 16 to 25, while our “40 year” estimates reflect survival from age 16 to 40. These values therefore represent conditional survival applying only to patients who had already survived to age 16, and should be interpreted in that context.

**Fig. 2:**
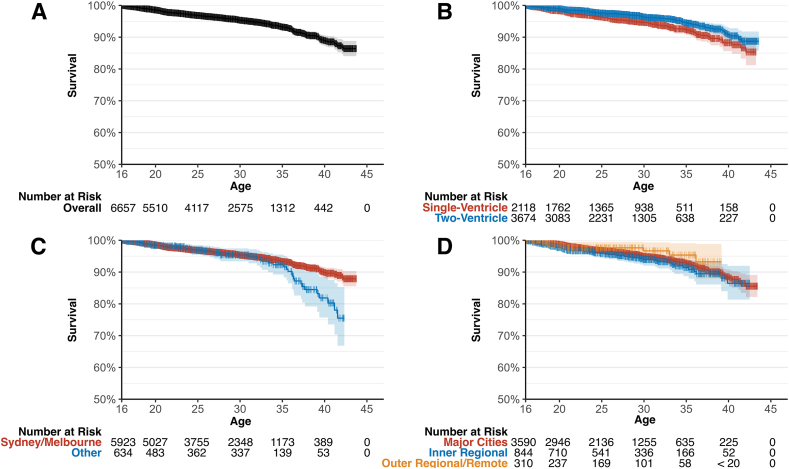
Kaplan–Meier curves for survival in 6557 severe congenital heart disease patients, A) overall; B) by single-ventricle versus two-ventricle physiology; C) by location of treatment centre; and D) by geographic remoteness of residence. Confidence intervals (95 %) displayed as a light-coloured band around the central survival curve line.

At 25 years of age, cumulative survival for the cohort was 96.9 % (95 % CI 96.4–97.3). By age 40, survival had declined to 89.0 % (95 % CI 87.3–90.6). Of those who died, the median age at death was 24 years (IQR 20–31). Fig. 2A shows the Kaplan–Meier survival curve for the entire cohort. Bearing in mind the known accuracy limitations of death certificates and hospital discharge summaries in the context of CHD [14,15], the top 5 most common documented causes of death were heart failure (n = 72; 23.0 %), cardiac arrest (n = 40; 12.8 %), pneumonia (n = 25; 8.0 %), sepsis (n = 19; 6.1 %), and respiratory failure (n = 17; 5.4 %). Notably, heart failure accounted for 31.1 % of single-ventricle patient deaths versus 16.3 % of two-ventricle patient deaths (chi-squared p = 0.009).

### 3.2 Single- versus two-ventricle

In our severe CHD cohort, 2118 patients (32 %) had single-ventricle physiology and 3674 (56 %) had two-ventricle physiology despite complex anatomy. There were 765 patients (12 %) whose physiology could not be confidently classified due to insufficient clinical information and who were therefore excluded from single-versus two-ventricle analysis. This group had broadly similar characteristics to the single- and two-ventricle groups; there were no significant differences in remoteness, treatment location or age at death between the subgroups (Table 4). Although the difference in median age (and interquartile ranges) at last follow-up was small (29 [23, 35] vs 27 [22, 32] years), the large sample size yielded a statistically significant difference (p < 0.001). Furthermore, there was a small difference in mean year of birth: 1994 for single-ventricle patients versus 1996 for two-ventricle patients (p < 0.001).The clinical significance of these small differences is likely limited.

**Table 4:** Cohort characteristics stratified by ventricular physiology. P values calculated for single-versus two-ventricle using chi-squared tests for categorical variables and Wilcoxon rank-sum tests for continuous variables. Demographics for patients unable to be classified by our algorithm (“Unknown”) also included for comparison.

Characteristic	Single-Ventricle	Two-Ventricle	p	*Unknown*
**Total**	2118 (32 %)	3674 (55 %)		*765 (11 %)*
**Sex**			0.6	
Male	1210 (57 %)	2071 (56 %)		*370 (48 %)*
Female	905 (43 %)	1593 (43 %)		*395 (52 %)*
Not Stated	3 (0.1 %)	10 (0.3 %)		*0 (0 %)*
**Clinic Location**			0.4	
Sydney/Melbourne	1913 (90 %)	3294 (90 %)		*716 (94 %)*
Other	205 (10 %)	380 (10 %)		*49 (6 %)*
**Remoteness**			0.9	
Major Cities	1146 (54 %)	1987 (54 %)		*457 (60 %)*
Inner Regional	269 (13 %)	482 (13 %)		*93 (12 %)*
Outer Regional/Remote	104 (5 %)	180 (5 %)		*26 (3 %)*
Multiple	425 (20 %)	274 (8 %)		*63 (8 %)*
Unknown	425 (20 %)	751 (20 %)		*126 (16 %)*
**Age**				
Age at Death	24 (19, 31)	24 (20, 31)	0.9	*22 (20, 31)*
Age at Last Follow-Up	29 (23, 35)	27 (22, 32)	<0.001	*29 (24, 34)*
**Year of Birth**	’94 (’88-’01)	’96 (’91-’01)	<0.001	*’94 (’89-’99)*

Overall, adults with single-ventricle severe CHD exhibited significantly worse survival than those with two-ventricle severe CHD (Fig. 2B). By age 25, cumulative survival for single-ventricle patients was 96.4 %, compared to 97.6 % for those with two-ventricle severe CHD. This gap widened with time: at age 40, estimated survival was only 88.2 % in the single-ventricle group versus 90.8 % in the two-ventricle group.

On Schoenfeld residuals testing, all covariates were found to meet the proportional hazards assumptions (all p > 0.05), though treatment location was borderline (p = 0.056). The hazard ratio for mortality associated with single-ventricle physiology was 1.46 (95 % CI 1.08–1.98; p = 0.014) when controlled for covariates as described.

### 3.3 Sydney/Melbourne versus other cities

Despite the fact that just 56 % of Australians live in the states New South Wales or Victoria, most of our cohort (n = 5923, 90 %) underwent treatment and follow-up in Sydney or Melbourne, the two largest cities in Australia, with a minority (n = 634, 10 %) being seen elsewhere. This is likely due to the fact that these clinics are the oldest, and have therefore collected the most data. There was no difference in the proportion of single-versus two-ventricle patients since in Sydney/Melbourne versus other cities (single-ventricle n = 1913/5923 [32 %] in Sydney/Melbourne, single-ventricle n = 205/634 [32 %] in other cities, chi-squared p = 0.45), but there was a difference in residential remoteness: while 76 % of patients seen in Sydney/Melbourne live in a major city and 6 % live in outer regional/remote areas, 70 % of patients seen in other cities live in a major city and 13 % live in outer regional/remote areas (chi-squared p < 0.001). This likely due to the fact that 74 % of the New South Wales residents and 79 % of Victoria residents live in a major city, versus 62 % for Queensland, the next largest state [16].

**Fig. 3:**
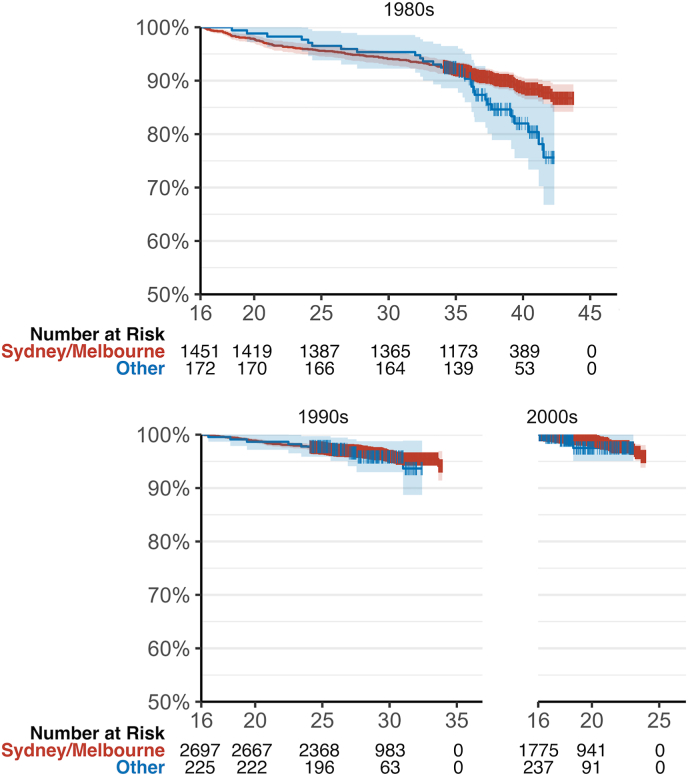
Kaplan–Meier curves for survival stratified by treatment location and patient birth decade. There is a significant difference in mortality between treatment in Sydney/Melbourne versus other cities for patients born in the 1980s (log-rank p = 0.028) but not for those born in the 1990s (p = 0.70) or 1990s (p = 0.60).

Patients seen in Sydney/Melbourne had significantly better survival than those seen elsewhere, especially from around age 35 (25-year survival 96.8 % vs 96.9 %; 40-year survival 89.9 % vs 81.9 %; p = 0.027, Fig. 2C). The covariate-adjusted hazard ratio for mortality of patients seen outside Sydney/Melbourne was 1.47 (95 % CI 0.98–2.21, p = 0.06). By way of a sensitivity analysis, we plotted survival curves stratified by birth decade (Fig. 3). It is clear visually and statistically that the mortality difference we see in the overall plot is limited to those patients born in the 1980s (p = 0.028). Log-rank tests show no significant difference in mortality for patients born in the 1990s (p = 0.70) and the 2000s (p = 0.60).

### 3.4 Major city versus regional/remote

Of the 6557 patients in the severe CHD cohort, 3590 (55 %) lived in a major city at last follow-up, 844 (13 %) lived in an inner regional area, and 310 (4.7 %) lived in an outer regional or remote area. There were 1302 patients (20 %) without a known address, and 511 patients (7.8 %) who lived in areas with multiple possible remoteness scores depending on their specific address, which was masked for privacy purposes. There were no significant differences in demographics between the groups, including the proportion of single-ventricle physiology (n = 1146/3590 [32 %] in Major Cities, 269/844 [32 %] in Inner Regional and 104/310 [34 %] in Outer Regional/Remote, chi-squared p = 0.93). There was, however, a significant difference in the proportion of patients treated in Sydney/Melbourne versus other cities, with 90 % of patients living in major cities seen in these centres, versus 78 % of those living rurally (p < 0.001), likely as a result of residential differences between the states as described in section 3.3 (Table 5). There was no difference in average age at death but there was a difference in age at last follow-up and year of birth, with inner regional patients 2 years older on average at last follow-up than those in major cities or outer regional/remote areas (p < 0.001), and those in Major Cities born one year earlier on average than those in inner regional or outer regional/remote areas (p < 0.001), though these differences are small and likely of limited clinical significance.

**Table 5:** Cohort characteristics stratified by residential remoteness.

Characteristic	Major Cities	Inner Regional	Outer Regional/Remote	p	*Multiple*	*Unknown*
**Total**	3590 (54 %)	844 (13 %)	310 (5 %)		*511 (8 %)*	*1302 (20 %)*
**Sex**				0.78		
Male	1976 (55 %)	447 (53 %)	173 (56 %)		*299 (59 %)*	*756 (58 %)*
Female	1608 (45 %)	396 (47 %)	137 (44 %)		*212 (41 %)*	*540 (41 %)*
Not Stated	3 (0.2 %)	1 (0.1 %)	0 (0 %)		*0 (0 %)*	*6 (0.5 %)*
**Physiology**				0.17		
Single-ventricle	1146 (32 %)	269 (32 %)	104 (34 %)		*174 (34 %)*	*425 (33 %)*
Two-ventricle	1987 (55 %)	482 (57 %)	180 (58 %)		*274 (54 %)*	*751 (58 %)*
Unknown	457 (13 %)	93 (11 %)	26 (8 %)		*63 (12 %)*	*126 (10 %)*
**Clinic Location**				<0.001		
Sydney/Melbourne	3225 (90 %)	754 (89 %)	242 (78 %)		*448 (88 %)*	*716 (94 %)*
Other	365 (10 %)	90 (11 %)	68 (22 %)		*63 (12 %)*	*49 (6 %)*
**Age**						
Age at Death	24 (20, 31)	22 (19, 30)	20 (19, 30)	0.3	*24 (20, 32)*	*25 (19, 33)*
Age at Last Follow-Up	27 (22, 33)	29 (23, 34)	26 (21, 33)	<0.001	*27 (21, 33)*	*31 (25, 36)*
**Year of Birth**	’96 (’90-’01)	’97 (’91-’03)	’97 (’91-’03)	<0.001	*’96 (’89-’02)*	*’92 (’88-’98)*

There was no significant difference in survival by residential location (log-rank p = 0.22; Fig. 2D) and no evidence of a trend relating remoteness and mortality (log-rank test for trend p = 0.41). At age 25, survival was 96.9 % in major cities versus 95.9 % in inner regional areas and 97.6 % in outer regional and remote areas. At age 40, survival was 88.5 % in major cities versus 86.5 % in inner regional areas and 93.2 % in outer regional and remote areas. The covariate-adjusted hazard ratio for mortality of patients in inner regional Australia versus major cities was 1.28 (95 % CI 0.39–1.50, p = 0.16); the hazard ratio for outer regional/remote Australia versus major cities was 0.76 (95 % CI 0.33–1.28, p = 0.42).

## 4 Discussion

This study provides a comprehensive, contemporary analysis of mortality in adults with severe congenital heart disease using a large national Registry. We found that patients with severe CHD who reach adulthood have on average an 89 % conditional probability of living to the age of 40. There were, however, significant differences in prognosis in single-versus two-ventricle hearts: single-ventricle physiology conferred a significantly increased risk of death in adulthood, even when compared to other forms of severe CHD. There was also a significantly better prognosis in Sydney or Melbourne, where specialist ACHD centres are well-established and advanced cardiothoracic surgery has been long available for many decades, as opposed to other Australian cities. On the other hand, we found no significant difference in survival between patients residing in major cities and those in regional or rural areas, despite well-documented urban/rural health disparities in cardiovascular disease.

An 89 % chance for a 16 year old with severe CHD to live to age 40 is in stark contrast to the general Australian population conditional survival rate of 98.7 % [17], highlighting a substantial mortality gap. Nonetheless, our results appear more favourable than those reported from other international cohorts. For example, the Dutch CONCOR registry found a 40-year survival rate for severe CHD of about 80 % (conditional on reaching adulthood), though their registry used the older Bethesda guidelines rather than the ESC guidelines for severity classification [5,7,8], and included patients from around the 1950s onwards. Likewise, a Swedish national analysis of 24,774 CHD patients alive at 18 demonstrated a 5-fold higher mortality risk in all CHD patients versus matched controls, which rose to over 7-fold in patients selected for “severe” lesions [18]. Reporting survival conditional on reaching age 16 has particular clinical value in Australia, where adult CHD clinicians typically begin caring for patients at this age. These estimates are therefore directly relevant for prognostication in clinic, and are more informative for patient (and parent) counselling than mortality statistics calculated from birth. Given that a recent study in Belgium found that survival to adulthood for newborns with CHD has increased from 81 % in the early 1970s to around 89 % for births in the early 1990s [3], survival conditional on reaching adulthood will have ongoing and increasing prognostic importance.

The most common documented causes of death in our registry (where available from linkage) were heart failure (23.0 % of deaths), cardiac arrest (12.8 %) and pneumonia (8.0 %). Notably, heart failure was the leading cause of death, consistent with multiple studies showing that heart failure accounts for around 20–40 % of deaths in ACHD and is more common as complexity increases [19,20].

The most notable novel finding of this study is that severe CHD patients with single-ventricle physiology face a significantly higher risk of mortality compared to patients with two-ventricle physiology. This analysis represents the first direct comparison of survival between single-ventricle and other complex CHD patients in adulthood. These results are in line with previous studies that have documented the long-term outcomes of Fontan patients [21,22] One recent nationwide study from Sweden found that individuals born with single-ventricle anatomy had a more than 50-fold higher risk of mortality compared to the general population, but did not compare mortality between single- and two-ventricle severe CHD patients [23]. Our study has the advantage of longer-term follow-up, comprehensive multi-centre enrolment, and head-to-head comparison with two-ventricle severe CHD in the same context.

This difference in outcomes is likely as a result of physiological sequelae of persistent cyanosis and palliative rather than corrective surgery, in “Fontan” patients. From a pathophysiological standpoint, the reasons for poorer survival in Fontan patients are well recognised. The absence of a sub-pulmonary ventricle leads to chronically elevated systemic venous pressure and decreased cardiac output, leading to a cascade of complications including ventricular dysfunction, arrhythmias, and liver and kidney disease, amongst others. Over time, these issues often culminate in Fontan “failure”—defined by a combination of declining exercise capacity, heart failure, and end-organ dysfunction—which has no easy remedy short of heart transplantation. Completion of the Fontan circulation (or failure to achieve it) is an important determinant of survival, but as this variable is incompletely captured in our administrative datasets, it was not modelled directly. In our cohort, heart failure was the leading cause of death and was disproportionately represented among single-ventricle patients (accounting for 31.1 % of single-ventricle patient deaths versus 16.3 % of two-ventricle patient deaths).

We also found significantly better adult survival for patients managed in Sydney or Melbourne compared to those in other cities, a difference which we isolated to patients in our registry born in the 1980s. This likely reflects historical and systemic advantages in these major centres. Sydney and Melbourne have long hosted the largest and oldest specialised ACHD services, with established multidisciplinary teams and earlier access to advanced interventions. For example, the first successful heart transplant program in Australia launched in Sydney in 1968, whereas Queensland's state transplant service did not commence until 1990 and Western Australia's not until 1995. Likewise, many complex congenital heart surgeries (such as the Fontan procedure for single-ventricle physiology) were first adopted in Australia at the major paediatric cardiac centres in Sydney and Melbourne during the late 1970s. The emergence of specialist ACHD hubs in other states may explain the disappearance of the divide between Sydney/Melbourne and other cities from the 1990s onward.

Unlike in many other countries, the unique “hub-and-spoke” model of ACHD care in Australia means that referral patterns are unlikely to represent a major source of bias: in Australia, patients and clinicians do not generally choose between centres, but are referred to a single ACHD clinic per state according their residence (e.g. New South Wales to Sydney, Victoria to Melbourne, Queensland to Brisbane), supported by more regular follow-up with local general practitioners. Unfortunately, socioeconomic status was not routinely collected in our database and may represent an uncontrolled confounder for our results. However, because Australian states have broadly similar levels of socioeconomic advantage and disadvantage, and because referrals are made based on state residence, we believe this is unlikely to have introduced substantial bias into our results. Differing severity of CHD between treatment centres is also unlikely to be a major confounder, as we have specifically selected patients who are all classed as “severe”. Though there may be differences in mortality between individual severe CHD lesions, we have no reason to suspect that patients born in the most populous states in Australia will have more serious lesions on average. Furthermore, although we are unable to determine the proportion of patients who were referred interstate specifically for treatment, the experience of our clinicians suggests this number is very low. Nonetheless, informative censoring due to interstate transfer cannot be entirely ruled out. As Sydney/Melbourne centres are older and more established, they are likely to have data on more patients from earlier eras in CHD care, which may artificially lower their survival rates. We were, however, able to control for birth decade in our analysis, finding in our sensitivity analysis that the difference between Sydney/Melbourne and other treating centres was isolated to the 1980s, the earliest decade in our study (Fig. 3). Ultimately, our data suggest that concentrating complex CHD care in well-resourced centres may confer benefits, though residual confounding cannot be excluded and interpretation is limited by the small size of the comparator group.

This finding is particularly pertinent in the context of our surprising null result that there was no difference in mortality between those living in the city and in the country. We had anticipated that patients with severe CHD living in outer regional or remote areas might have worse outcomes, possibly due to reduced access to specialised ACHD care, later referral for advanced therapies, or socioeconomic disadvantage. Instead, as previously found by our group for CHD patients post-Fontan or Tetralogy of Fallot repair [24], no geographical mortality difference was detected. This contrasts with evidence from general cardiovascular disease populations [25,26], and even in a large CHD population in the USA [27].

Several particularities of the Australian context could be contributing to this null finding. Firstly, ACHD care in Australia is highly centralised in capital cities, meaning that patients with severe CHD who live rurally are seen in the same advanced quaternary hospitals as city-dwellers. Secondly, in recent years, there has been an expansion of outreach clinics staffed by the same specialist cardiologists as in the cities, bringing high-level care coordination directly to patients who might otherwise face insurmountable travel difficulties. Thirdly, there could be an element of survival bias in our findings, with patients with worse CHD requiring more frequent follow-up relocating to the city, while patients with less impactful disease are able to remain in regional or remote areas, negating a potential urban-rural health differential. Nonetheless, if there is indeed parity in outcomes by geography, it would be an encouraging indication that the “hub-and-spoke” model of ACHD care implemented in Australia has been successful, and evidence that the networks and resources that facilitate this model should continue to receive support.

Our study has other limitations not previously mentioned. Firstly, by design, we focused on all-cause mortality as an endpoint and did not delve deeply into morbidity measures, which might reveal disparities where mortality did not. Our registry is in the process of integrating such data and future studies will address this gap. Secondly, the accuracy of our findings on mortality is dependent on linkage to the Australian National Death Index. While this registry is high-quality and comprehensive when it comes to vital status, our group has previously found that cause-of-death coding can be imprecise or incomplete in CHD [14,15], so our cause-of-death findings should be interpreted cautiously. Thirdly, though our analysis was based on up-to-date severity classification guidelines and was novel in its automatic selection of severe CHD patients alone, there remain well-known differences in mortality risk within the “severe” category which we were unable to characterise in this study. Our broad overview of mortality in this large population did not permit a lesion-by-lesion or surgery-by-surgery analysis, and we did not have access to data on perioperative complications. Our algorithm did, however, incorporate surgical history into classification: for example, transposition of the great arteries with an arterial switch procedure is categorised as moderate CHD, whereas with an atrial switch it is categorised as severe, ensuring that only surgical pathways associated with severe outcomes were included. Though this is a problem inherent to the study of such a heterogenous set of lesions, it is our hope that the growth of large national (or international) registries such as ours will eventually allow for more granular lesion-specific analysis, especially as we look to expand the coverage of our database to New Zealand. Finally, we did not have measures of functional status available in our database, though we are now enrolling patients for a sub-study that will provide much more granular data, including functional status, psychological outcomes, objective stress testing and echocardiography, which will enhance future prognostic modelling.

In conclusion, in this report, we have quantified the mortality burden among adults with severe CHD. Adults with severe CHD face significantly higher mortality rates than the general population, particularly those with single-ventricle physiology.

## CRediT authorship contribution statement

**Jason Chami:** Writing – review & editing, Writing – original draft, Visualization, Validation, Software, Resources, Project administration, Methodology, Investigation, Formal analysis, Data curation, Conceptualization. **Calum Nicholson:** Writing – review & editing, Software, Project administration, Data curation. **David Baker:** Writing – review & editing, Supervision, Resources, Project administration, Conceptualization. **Rachael Cordina:** Writing – review & editing, Supervision, Resources, Conceptualization. **Geoff Strange:** Writing – review & editing, Supervision, Resources, Conceptualization. **David S. Celermajer:** Writing – review & editing, Supervision, Resources, Project administration, Methodology, Investigation, Funding acquisition, Data curation, Conceptualization.

## Grant support

The development of a comprehensive Australia and New Zealand CHD Registry was initially funded by philanthropic donations from HeartKids Australia. Additional funding has been provided by an Australian Department of Health grant through the Medical Research Future Fund, grant code is ARGCHDG0000028.

## Declaration of competing interest

DSC reports financial support provided by 10.13039/100008131HeartKids Australia Inc. and by the Australian Government Department of Health, Disability and Ageing. DSC and RC serve on the IJCCHD Editorial Board, but had no involvement with the handling of this paper. There are no other known competing financial interests or personal relationships that could have appeared to influence the work reported in this paper.

## References

[1] Brida M., Gatzoulis M.A. (2019). Adult congenital heart disease: past, present and future. Acta Paediatr.

[2] Liu A. (2023). Changing epidemiology of congenital heart disease: effect on outcomes and quality of care in adults. Nat Rev Cardiol.

[3] Moons P., Bovijn L., Budts W., Belmans A., Gewillig M. (2010). Temporal trends in survival to adulthood among patients born with congenital heart disease from 1970 to 1992 in Belgium. Circulation.

[4] Marelli A.J. (2014). Lifetime Prevalence of congenital heart disease in the general population from 2000 to 2010. Circulation.

[5] Van der Bom T. (2015). Contemporary survival of adults with congenital heart disease. Heart.

[6] Müller M.J. (2022). Morbidity and mortality in adults with congenital heart defects in the third and fourth life decade. Clin Res Cardiol.

[7] Baumgartner H. (2021). 2020 ESC guidelines for the management of adult congenital heart disease: the task force for the management of adult congenital heart disease of the European society of cardiology (ESC). Endorsed by: Association for European Paediatric and congenital Cardiology (AEPC), International Society for Adult Congenital Heart Disease (ISACHD). Eur Heart J.

[8] Warnes C.A. (2001). Task Force 1: the changing profile of congenital heart disease in adult life. J Am Coll Cardiol.

[9] Chami J. (2023). Algorithmic complexity stratification for congenital heart disease patients. Int J Cardiol Congenit Heart Dis.

[10] Chami J., Nicholson C., Strange G., Cordina R., Celermajer D.S. (2021). National and regional registries for congenital heart diseases: strengths, weaknesses and opportunities. Int J Cardiol.

[11] Nicholson C. (2024). A national Australian congenital Heart Disease registry; methods and initial results. Int J Cardiol Congenit Heart Dis.

[12] Chami J. (2024). Improved complexity stratification in congenital heart disease; the impact of including procedural data on accuracy and reliability. Int J Cardiol Congenit Heart Dis.

[13] Franklin R. (2002). The European Paediatric Cardiac Code Long list: structure and function — the first revision. Cardiol Young - CARDIOL YOUNG.

[14] Chami J. (2022). High error rates in coding causes of death in adults with congenital heart disease. JACC Adv.

[15] Chami J. (2022). Hospital discharge codes and substantial underreporting of congenital heart disease. Int J Cardiol Congenit Heart Dis.

[16] Australian Bureau of Statistics (2023). Regional population. https://www.abs.gov.au/statistics/people/population/regional-population/latest-release.

[17] Australian Institute of Health and Welfare (2025). Deaths in Australia. https://www.aihw.gov.au/reports/life-expectancy-deaths/deaths-in-australia/contents/life-expectancy.

[18] Fazlinovic S. (2025). Survival trends of adults with congenital heart disease after heart surgery in Sweden. J Thorac Cardiovasc Surg.

[19] Verheugt C.L. (2010). Mortality in adult congenital heart disease. Eur Heart J.

[20] Diller G.-P. (2015). Survival prospects and circumstances of death in contemporary adult congenital heart disease patients under Follow-Up at a large tertiary centre. Circulation.

[21] d'Udekem Y. (2014). Redefining expectations of long-term survival after the Fontan procedure. Circulation.

[22] Khairy P. (2008). Long-term survival, modes of death, and predictors of mortality in patients with Fontan surgery. Circulation.

[23] Öztürk A.-G. (2024). Long-term survival in patients with univentricular heart: a nationwide, register-based cohort study. Int J Cardiol Congenit Heart Dis.

[24] Lloyd L. (2025). Excellent medium to long term outcomes after cardiac surgery for moderate and complex congenital heart disease, regardless of geographic location. Int J Cardiol Congenit Heart Dis.

[25] Australian Institute of Health and Welfare (2019). Cardiovascular disease. https://www.aihw.gov.au/reports/heart-stroke-vascular-disease/cardiovascular-health-compendium/contents/deaths-from-cardiovascular-disease.

[26] Tan E.J. (2021). Trends in ischaemic heart disease in Australia, 2001-2015: a comparison of urban and rural populations. Heart Lung Circ.

[27] Abdul Mannan Khan Minhas M.D. (2022). Rural-Urban Trends in Congenital Heart Disease-Related Mortality in the United States, 1999 to 2019. JACC Adv.

